# Mitral annular dynamics are influenced by left ventricular load and contractility in an acute animal model

**DOI:** 10.14814/phy2.15665

**Published:** 2023-04-16

**Authors:** Robert Matongo Persson, Hans Martin Dahl Aguilera, John‐Peder Escobar Kvitting, Ketil Grong, Victorien Emile Prot, Pirjo‐Riitta Salminen, Bård Svenheim, Anita Leiknes, Lodve Stangeland, Rune Haaverstad, Stig Urheim

**Affiliations:** ^1^ Department of Heart Disease Haukeland University Hospital Bergen Norway; ^2^ Department of Clinical Science, Faculty of Medicine University of Bergen Bergen Norway; ^3^ Department of Structural Engineering, Faculty of Engineering Science The Norwegian University of Science and Technology Trondheim Norway; ^4^ Department of Cardiothoracic Surgery Oslo University Hospital, Rikshospitalet Oslo Norway; ^5^ Institute of Clinical Medicine University of Oslo Oslo Norway

**Keywords:** mitral annular dynamics, mitral regurgitation, mitral valve annulus, mitral valve physiology, sonomicrometry

## Abstract

The purpose of this study was to investigate the effects of loading conditions and left ventricular (LV) contractility on mitral annular dynamics. In 10 anesthetized pigs, eight piezoelectric transducers were implanted equidistantly around the mitral annulus. High‐fidelity catheters measured left ventricular pressures and the slope of the end‐systolic pressure‐volume relationship (*E*_es_) determined LV contractility. Adjustments of pre‐ and afterload were done by constriction of the inferior caval vein and occlusion of the descending aorta. Mitral annulus area indexed to body surface area (MAA_i_), annular circularity index (ACI), and non‐planarity angle (NPA) were calculated by computational analysis. MAA_i_ was more dynamic in response to loading interventions than ACI and NPA. However, MAA_i_ maximal cyclical reduction (−Δr) and average deformational velocity (−$\bar{v}$) did not change accordingly (*p =* 0.31 and *p =* 0.22). Reduced *E*_es_ was associated to attenuation in MAA_i_‐Δr and MAA_i_‐$\bar{v}$ (*r* ^2^ = 0.744; *p* = 0.001 and *r* ^2^ = 0.467; *p* = 0.029). In conclusion, increased cardiac load and reduced LV contractility may cause deterioration of mitral annular dynamics, likely impairing coaptation and increasing susceptibility to valvular incompetence.

## Graphical abstract

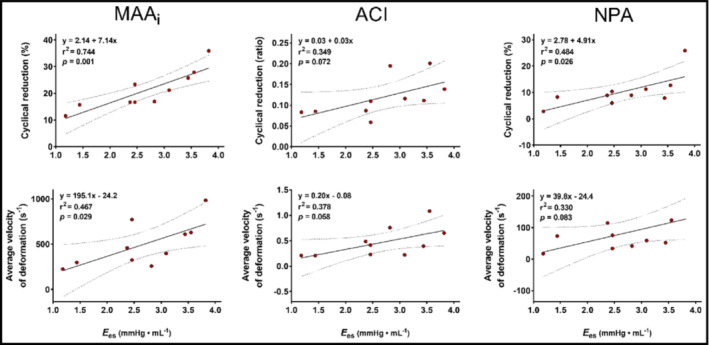

**NEW & NOTEWORTHY**

In a large animal model applying a hybrid methodology with sonomicrometer array localization and computational analysis, we determine that mitral annular dynamics are load‐ and contractility‐dependent. These findings may provide explanations for pathological mechanisms in mitral regurgitation and premises for novel treatment strategies.

## 1 INTRODUCTION

The mitral annulus (MA) is an integral part of the mitral valve apparatus. During the cardiac cycle, the MA undergoes a geometric deformation from circular to ellipsoid with subsequent reduction in mitral annular area, as well as three‐dimensional folding and flattening of the mitral annulus saddle shape. The alterations of annular geometry through the cardiac cycle are thought to reduce leaflet stress and sustain coaptation in ventricular systole, and facilitate ventricular filling in diastole (Silbiger, 2012).

Mitral regurgitation (MR) is commonly associated with abnormal annular geometry, and correcting for annular dilatation is a fundamental principle of mitral valve repair (Carpentier et al., 2010). Because the MA does not possess any intrinsic contractile properties, its dynamic motion is assumed to be secondary to ventricular and atrial contraction (Silbiger & Bazaz, 2020). In chronic disease with ventricular remodeling, the left ventricular (LV) function has a significant effect on the mitral valve (Asgar et al., 2015). The acute coupling of ventricular mechanics and loading conditions to the dynamic motion of the MA, however, has not been thoroughly described. The grade of MR can be influenced by hemodynamic changes, causing exercise intolerance and precipitating hospitalization with acute pulmonary edema (Bertrand et al., 2017; Piérard & Lancellotti, 2004). An accurate description of these acute relationships may provide insights into the pathophysiology of MR and guide treatment strategies.

Previous experimental research using different animal models has provided a framework for understanding normal and pathological mitral valve function (Rausch et al., 2011; Silbiger & Bazaz, 2020). In this study, we aimed to investigate the acute effects of load alterations and LV contractility on MA geometry and deformation utilizing an in vivo experimental porcine model in combination with computational data analysis.

## 2 METHODS

### 2.1 Experimental protocol

The study was performed in compliance with the European Communities Council Directive of 2010 (63/EU) and approved by the Norwegian State Commission for Laboratory Animals (Project ID: 14687). Ten Norwegian landrace pigs (*Sus scrofa domesticus*) of either sex (average weight 58 (SD 5) kg) were used. As previously described, the premedication and anesthetic protocol has been evaluated for safe use of neuromuscular blocking agents and with minor influence on cardiac and hemodynamic function (Fanneløp et al., 2004). Specifically, the animals were premedicated with an intramuscular injection of ketamine (20 mg kg^−1^), diazepam (10 mg), and atropine (1 mg) and mask‐ventilated with 3% isoflurane until induction of intravenous anesthesia. Two intravenous access lines were established on which the animal was administered a combination of fentanyl (0.02 mg kg^−1^), midazolam (0.3 mg kg^−1^), pentobarbital (15 mg kg^−1^), and pancoronium (0.063 mg kg^−1^). The animals were thereafter mechanically ventilated with a mixture of nitrous oxide (57%–58%) and oxygen (Primus, Drägerwerk) through a tracheotomy and monitored by regular blood gases, surface electrocardiogram (ECG), pulse oximetry, rectal temperature, and diuresis. Anesthesia was maintained with continuous intravenous infusion of fentanyl (0.02 mg kg^−1^ h^−1^), midazolam (0.3 mg kg^−1^ h^−1^), pancuronium (0.2 mg kg^−1^ h^−1^), and pentobarbital (4 mg kg^−1^ h^−1^). Fluid was substituted with Ringer's acetate at 15 mL kg^−1^ h^−1^ throughout the experiment, and bolus was given after weaning from cardiopulmonary bypass.

In dorsal recumbency, the heart was exposed through median sternotomy and suspended in a pericardial cradle. After partial heparinization (125 IU kg^−1^), pre‐calibrated micromanometer‐tipped pressure transducers (MPC‐500, Miller Instruments Inc.) were placed in the left ventricle through the apex of the heart and in the left atrium through the left atrial appendage together with a fluid‐filled catheter for zero‐referencing. A second fluid‐filled catheter was placed in the descending aorta through the left carotid artery for monitoring purposes. Endovascular occlusive balloon catheters were implanted in the descending aorta through the femoral artery, and the inferior caval vein (IVC) was snared with a surgical snugger. Five two‐millimeter piezoelectric transducers (Sonometrics Corp.) were placed epicardially; one at the apex of the heart and four equidistantly at the ventricular equatorial plane. After full heparinization (500 IU kg^−1^), cardiopulmonary bypass was prepared with a 23‐Fr two‐stage venous cannula through the right atrium and 17‐Fr arterial cannula through the femoral artery. While on cardiopulmonary bypass the ascending aorta was cross‐clamped, and the heart arrested with antegrade cold crystalloid cardioplegia (St. Thomas' Solution No 2). The mitral valve was exposed through a left atriotomy, and eight piezoelectric transducers were sutured to the mitral annulus. The left atrium was closed, and the animal weaned from cardiopulmonary bypass. Mean cardiopulmonary bypass and aortic cross‐clamp times were 98 (SD 15) and 55 (SD 15) min, respectively.

### 2.2 Data acquisition

Transducer wires were connected to an external ultrasound transceiver and acquisition system (Sonometrics Corp.). After weaning from cardiopulmonary bypass, sonomicrometer array localization was used to determine the transducer positions at 8.53 ms intervals, which gives accurate measurements within a 1% error margin and a theoretical spatial resolution of 0.024 mm (Gorman 3rd et al., 1996). Sonomicrometry measurements were obtained after ventilation was suspended in the end‐expirium followed by epicardial three‐dimensional echocardiographic recordings (Vivid E9, GE Vingmed Ultrasound, Horten, Norway). Registrations were acquired at baseline, after transient inferior caval constriction (ICC), and after endovascular balloon occlusion of the descending aorta (AO), in this order. Animals were allowed adequate time to stabilize hemodynamically (5–10 min) between loading interventions. All registrations were in sinus rhythm in absence of mitral regurgitation. Post‐mortem inspection confirmed crystal positions in all animals.

### 2.3 Data analysis

All hemodynamic and geometric data were processed as a mean of three consecutive heartbeats. Measurements were calculated at a stable baseline situation, after caval constriction at 20% decrease of left ventricular end‐diastolic pressure (LV‐EDP) and immediately after plateau of peak systolic left ventricular pressures (LV‐SP_max_) following aortic occlusion. To adjust for weight differences, volumes and area measurements were indexed to body surface area (BSA) calculated with the Kelley formula as BSA (m^2^) = 734⋅BW_kg_^0.656^ (Kelley et al., 1973).

Left ventricular volume was estimated through a two‐axis ellipsoid model (*V* = $\frac{\pi }{6}D$ ^2^⋅*L*) (Mercier et al., 1982), where *D* and *L* represent the short axis at the equatorial anteroposterior diameter and the long apicobasal axis of the LV, respectively. Distances were obtained from corresponding transducer pairs and corrected for apical, end‐diastolic (ED), and end‐systolic (ES) wall thickness in the three‐dimensional echocardiography recordings (EchoPAC version 112; GE Healthcare). ED was defined as the point at which there was a rise in the first derivative of left ventricular pressure (*d*P/*d*t), coinciding with the right lower corner of the pressure‐volume loop. Correspondingly, ES was set at the minimum LV volume coinciding with the left upper corner of the pressure‐volume loop. The load‐independent index of the left ventricular contractility was determined by the slope (*E*_es_) of a linear regression line through the end‐systolic pressure‐volume relationship of 10 sequential heartbeats during ICC.

**FIGURE 1:**
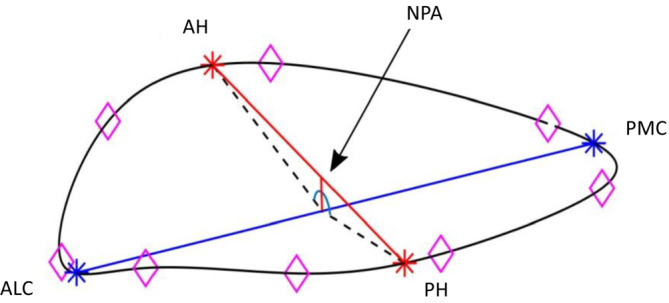
Computational reconstruction of the mitral annulus with cubic spline interpolation through transducer coordinates (pink diamond shape) and principal component analysis for geometric calculations. Red and blue axes designate septolateral and intercommissural distances, respectively. AH and PH, anterior and posterior saddle horn; ALC and PMC, anterolateral and posteromedial commissure; NPA, non‐planarity angle.

To determine positional data, the Cartesian coordinates were calculated as a function of time (CardioSoft and SonoXYZ; Sonometrics Corp.) and imported into MATLAB (MATLAB version 2021a; The MathWorks) where the mitral annulus was reconstructed by performing a cubic spline interpolation through the coordinate points. The mitral annular area (MAA) was obtained by projecting the interpolated spline onto its corresponding least squares plane and calculating the 2D area. Principal component analysis (PCA) was then performed on the annular spline to make geometric calculations independent of transducer positions. The principal axes were designated to intersect the septolateral (SL) and commissural dimensions. The intercommissural width (ICW) was expressed as the longest distance between the commisures and was calculated by acquiring the annular points that intersected the first principal axis. Likewise, the SL distance was expressed as the distance separating the anterior and posterior horn of the annulus by defining the annular splines intersection of the second principal axis (Appendix A). Annular circularity index (ACI) was calculated as a ratio between these orthogonal diameters (SL/ICW). The non‐planarity angle (NPA) was calculated as the angle between the anterior and posterior annular horn at the juncture of the SL and CW axes. The computational technique with annular spline interpolation and PCA is illustrated in Figure 1.

To describe deformational kinematics to mitral annular geometry, maximum cyclical reduction (Δr) was determined as the absolute (ACI) or percentual (MAA and NPA) difference between the maximum and minimum measurement for each heartbeat. To determine the velocity of geometric deformation ($\bar{v}$), an average of the first derivative of time was calculated from the descending segments of cyclical deformation. For the presentation of cyclical alterations of geometric variables, data were transformed with linear temporal interpolation and normalized for time. The fractional change of each annular metric was calculated as the percentual difference in relation to the end‐systolic geometric configuration of the previous heartbeat.

### 2.4 Statistical analysis

Data were analyzed using SigmaPlot v.14.5 (Systat Software Inc.). After testing for normality (Shapiro–Wilk test) and equal variance (Brown‐Forsythe test) a One‐way Analysis of Variance for repeated measurements or Friedman Repeated Measures Analysis on Ranks was used whenever appropriate to compare baseline versus ICC and AO. If significant, post hoc multiple comparisons were done with Holm‐Sidak tests or Dunn's tests. For comparison of mitral annular deformational kinematics to left ventricular contractile index, simple linear regression analysis was performed with 95% confidence interval. All values are given as mean ± SE or median (1st quartile; 3rd quartile) unless otherwise noted. *p*‐values were considered significant when <0.05.

## 3 RESULTS

### 3.1 Hemodynamic responses to load interventions

The hemodynamic responses to transient ICC and AO can be viewed in Table 1. Most notably, ICC caused a reduction of left ventricular end‐diastolic, peak systolic‐ and end‐systolic pressures (LV‐EDP, LV‐SP_max_, and LV‐ESP) as compared to baseline (*p* = 0.025, *p* < 0.001, and *p* < 0.001, respectively), with a subsequent reduction in indexed end‐diastolic volume and stroke volume (LV‐EDV_i_ and LV‐SV_i_) (*p* < 0.001 for both). Following AO, heart rate decreased (*p* = 0.008), and left ventricular pressures significantly increased (*p* < 0.001 for all), also increasing indexed end‐systolic volume (LV‐ESV_i_) (*p* = 0.007).

**TABLE 1:** Left ventricular hemodynamic variables at baseline, inferior caval constriction (ICC), and aortic occlusion (AO).

Variable	Baseline	ICC	AO	RM‐ANOVA
HR (beats min^−1^)	135 ± 10	135 ± 10	123 ± 8*	*p* = 0.006
LV‐EDP (mmHg)	9.8 ± 0.7	7.9 ± 0.5*	13.2 ± 1.2*	*p* < 0.001
LV‐SP_max_ (mmHg)	76 ± 5	61 ± 6*	111 ± 6*	*p* < 0.001
LV‐ESP (mmHg)	67 ± 6	52 ± 6*	105 ± 6*	*p* < 0.001
*d*P/*d*t_max_ (mmHg s^−1^)	1194 ± 172	968 ± 143*	1427 ± 203*	*p* < 0.001
*d*P/*d*t_min_ (mmHg s^−1^)	−871 ± 104	−651 ± 95*	−1267 ± 126*	*p* < 0.001
LV‐EDV_i_ (mL m^−2^)	113 ± 7	100 ± 6*	116 ± 7	*p* < 0.001
LV‐ESV_i_ (mL m^−2^)	72 ± 5	69 ± 6	78 ± 5*	*p* < 0.001
LV‐SV_i_ (mL m^−2^)	41 ± 3	31 ± 3*	39 ± 2	*p* < 0.001
LV‐EF (%)	38 (33; 41)	33 (27; 36)*	34 (32; 36)	*p* = 0.003
CI (L min^−1^ m^−2^)	5.5 ± 0.5	4.0 ± 0.4*	4.7 ± 0.4*	*p* = 0.001
*E*_es_ (mmHg mL^−1^)	2.66 ± 0.27	–	–	n.a.

*Note*: Values are mean ± SE or median (1st quartile; 3rd quartile); *n* = 10.

Abbreviations: HR, heart rate; LV, left ventricle; EDP, end‐diastolic pressure; SP_max_, peak systolic pressure; ESP, end‐systolic pressure; *d*P/*d*t_max_ and *d*P/*d*t_min_, maximum and minimum of the first derivative of left ventricular pressure; EDV and ESV, end‐diastolic and end‐systolic volumes; SV, stroke volume; _i_, value indexed for body surface area; EF, ejection fraction; CI, cardiac index; *E*_es_, slope of end‐systolic pressure‐volume relationship; *p*, *p*‐values for one‐way repeated measurement ANOVA or Friedman Repeated Measures Analysis of Variance on Ranks; n.a., not applicable.

* Significantly different from baseline with multiple contrast tests.

### 3.2 Influence of load on mitral annular geometry and deformation

**FIGURE 2:**
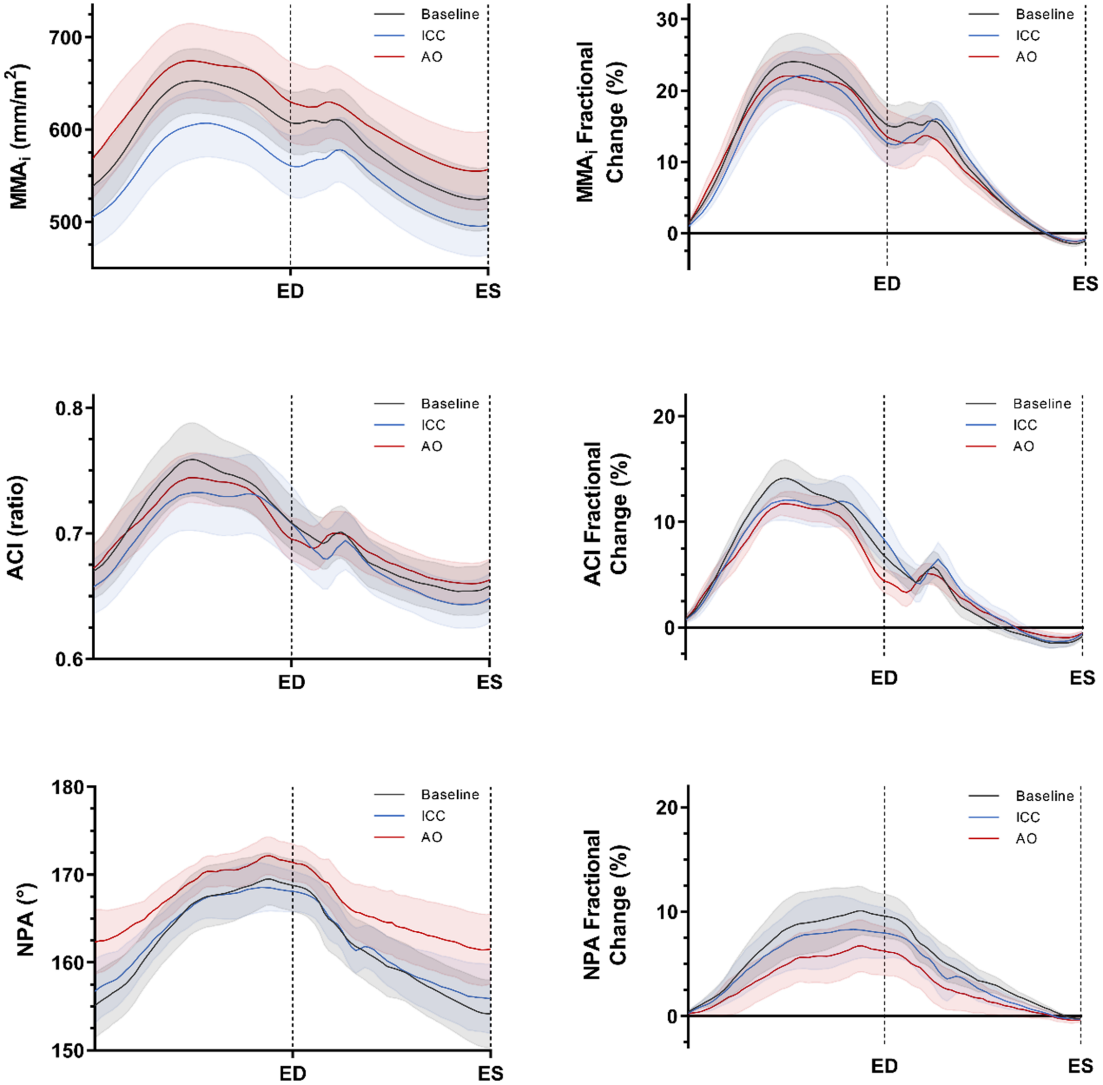
Cyclical deformation and fractional change for each geometric variable of mitral annulus for baseline, transient inferior caval constriction (ICC), and aortic occlusion (AO). Values are mean ± SE; *n* = 10. ED and ES, = delineators for end‐diastole and end‐systole; MAA_i_, indexed mitral annular area; ACI, annular circularity index; NPA, non‐planarity angle.

The cyclical deformation and fractional change for each mitral annular geometric variable during baseline, ICC, and AO are illustrated in Figure 2. Mitral annular dimensions and annular deformational kinematics at baseline and in response to ICC and AO can be viewed in Table 2. As compared to baseline, MA area indexed to body surface area (MAA_i_) decreased significantly in response to ICC at ED and SP_max_ (*p =* 0.013 and *p* = 0.008), and increased following aortic occlusion at ED, SP_max_, and ES (*p* = 0.008, *p* = 0.037, and *p* = 0.002). However, dynamic changes to MAA_i_ expressed as cyclical reduction (MAA_i_‐Δr) and average velocity of deformation (MAA_i_‐$\bar{v}$) did not change significantly with interventions to left ventricular load.

**TABLE 2:** Mitral annular geometric and deformational kinematics at baseline, inferior caval constriction (ICC), and aortic occlusion (AO).

Variable	Baseline	ICC	AO	RM‐ANOVA
MAA_i_‐ED (mm^2^ m^−2^)	607 ± 33	560 ± 33*	663 ± 49*	*p* < 0.001
MAA_i_‐SP_max_ (mm^2^ m^−2^)	605 ± 35	573 ± 36*	626 ± 40*	*p* < 0.001
MAA_i_‐ES (mm^2^ m^−2^)	525 ± 34	495 ± 32	587 ± 49*	*p* < 0.001
MAA_i_‐Δr (%)	21.2 ± 2.3	19.5 ± 2.3	19.6 ± 2.3	*p* = 0.31
MMA_i_‐$\bar{v}$ (s^−1^)	495 ± 78	439 ± 82	429 ± 67	*p* = 0.22
ACI‐ED (ratio)	0.71 ± 0.02	0.71 ± 0.03	0.70 ± 0.02	*p* = 0.73
ACI‐SP_max_ (ratio)	0.70 ± 0.02	0.69 ± 0.02	0.71 ± 0.02	*p* = 0.37
ACI‐ES (ratio)	0.66 ± 0.02	0.65 ± 0.02	0.66 ± 0.02	*p* = 0.30
ACI‐Δr (ratio)	0.12 ± 0.01	0.11 ± 0.02	0.10 ± 0.01	*p* = 0.105
ACI‐$\bar{v}$ (s^−1^)	0.40 (0.22; 0.68)	0.38 (0.20; 0.74)	0.33 (0.19; 0.48)	*p* = 0.27
NPA‐ED (^o^)	169 ± 3	168 ± 2	171 ± 2	*p* = 0.065
NPA‐SP_max_ (^o^)	162 ± 3	162 ± 3	166 ± 3*	*p* = 0.008
NPA‐ES (^o^)	155 ± 4	156 ± 4	162 ± 4*	*p* = 0.031
NPA‐Δr (%)	10.3 ± 1.9	10.0 ± 2.1	8.4 ± 2.0	*p* = 0.073
NPA‐$\bar{v}$ (s^−1^)	81 ± 19	75 ± 21	59 ± 17*	*p =* 0.015

*Note*: Values are mean ± SE or median (1st quartile; 3rd quartile); *n* = 10.

Abbreviations: ‐ED, ‐ES and ‐SP_max_, delineators for end‐diastole, end‐systole, and peak systolic left ventricular pressure; Δ*r*, maximal cyclical reduction (i.e., difference between the maximum and minimum value for each cardiac cycle); $\bar{v}$‐, average velocity of cyclical deformation; MAA_i_, indexed mitral annular area; ACI, annular circularity index; NPA, non‐planarity angle. *p*, *p*‐values for one‐way repeated measurement ANOVA or Friedman Repeated Measures Analysis of Variance on Ranks.

* Significantly different from baseline with multiple contrast tests.

ACI remained essentially unaffected by loading interventions both in absolute values at the designated cyclic intervals (ED, SP_max_, and ES), and in terms of annular kinematics (ACI‐Δr and ACI‐$\bar{v}$). The NPA was not altered significantly during ICC but was significantly increased at SP_max_ and ES after aortic occlusion (*p =* 0.021 and *p =* 0.029). In response to AO, the average velocity of deformation (NPA‐$\bar{v}$) was significantly attenuated as compared to baseline (*p =* 0.011). However, the percentual cyclical reduction of NPA (NPA‐Δr) did not change significantly with interventions to LV load.

### 3.3 Influence of contractility on mitral annular deformation

**FIGURE 3:**
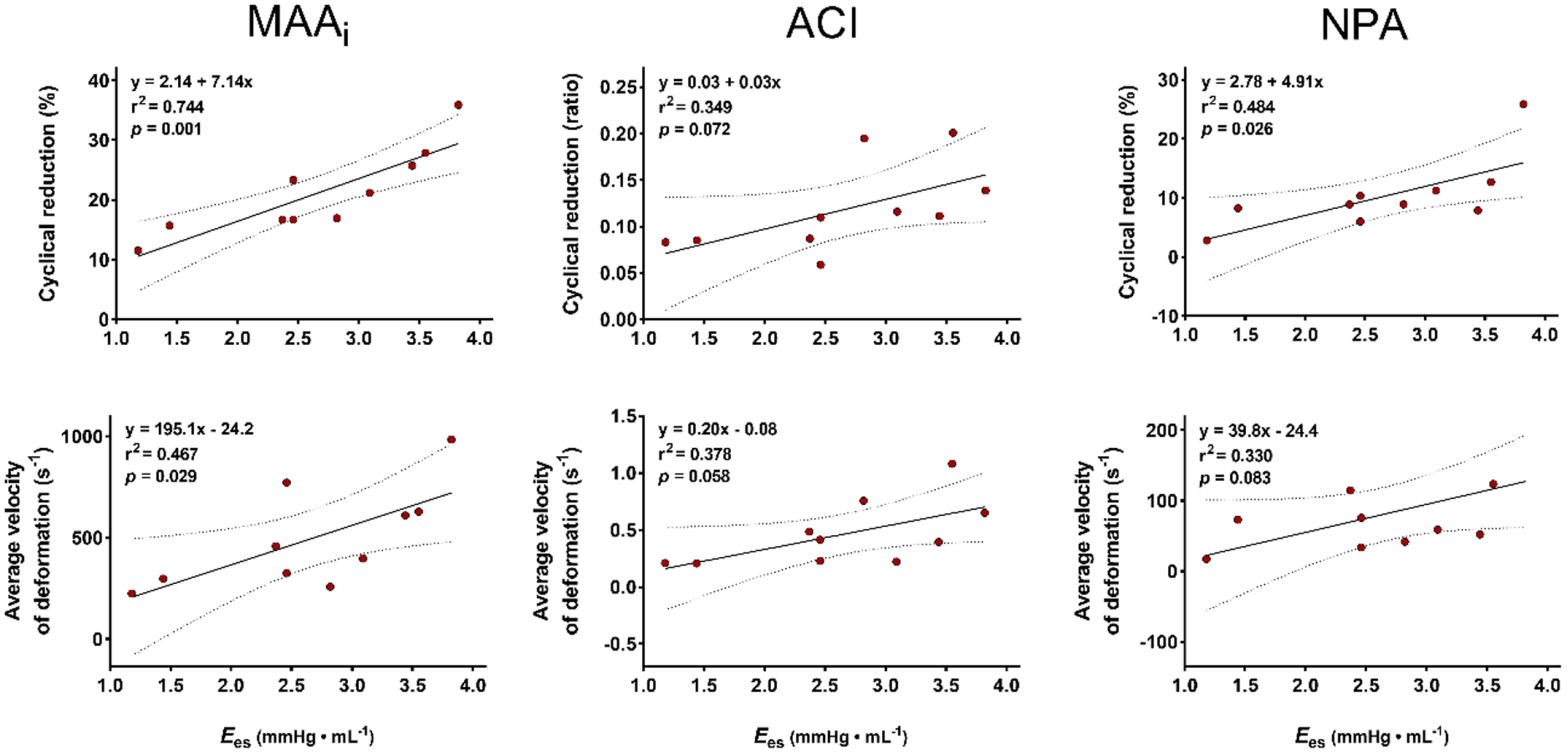
Scatterplot and linear regression analysis with 95% confidence interval correlating mitral annular deformational kinematics to left ventricular contractility; *n* = 10. MAA_i_, indexed mitral annular area; ACI, annular circularity index; NPA, non‐planarity angle; *E*_es_, slope of end‐systolic pressure‐volume relationship.

Figure 3 demonstrates linear regression analysis between LV contractile index (*E*_es_) and kinematics of annular deformation. MAA_i_‐Δr and MAA_i_‐$\bar{v}\;$ was significantly correlated to *E*_es_ (*r* ^2^ = 0.744; *p* = 0.001 and *r* ^2^ = 0.467; *p* = 0.029), with reduced contractility associated to attenuation in maximum percentual reduction and reduced velocity of deformation in MAA_i_. NPA‐Δr showed significant positive correlation to *E*_es_ (*r* ^2^ = 0.484; *p* = 0.026), while no significant correlation was seen with NPA‐$\bar{v}$ (*r* ^2^ = 0.330; *p* = 0.083). ACI was not significantly correlated to *E*_es_ either in cyclical reduction or velocity of deformation (*r* ^2^ = 0.349; *p* = 0.072 and *r* ^2^ = 0.378; *p* = 0.058).

## 4 DISCUSSION

### 4.1 Experimental results

The present study demonstrates that in an experimental large animal model, mitral annular dynamics are both load‐ and contractility‐dependent. More specifically, the mitral annular area appears more vulnerable to acute changes in load than annular circularity and saddle shape (Table 2). Following an increase in ventricular afterload, the annular saddle shape is flattened, and the area of the mitral annulus is symmetrically enlarged through the cardiac cycle (Figure 2). Because the magnitude of annular area reduction is proportionately unaltered with annular enlargement, the mitral leaflets are forced to coapt over a larger pressurized orifice. Taking into consideration increased ventricular pressures succeeding aortic occlusion, the response of annular geometry and kinematics to an increase in load may significantly increase tethering forces and reduce the leaflet closing forces during ventricular systole.

Furthermore, we demonstrate an association between left ventricular contractility and annular deformation (Figure 3). Most notably, cyclical reduction of the mitral annulus area is attenuated with reduced contractility, also reducing the efficacy of valvular closure. Independently or in combination, these findings may explain the individual susceptibility of valvular incompetence to a sudden increase in afterload and/or decrease in LV contractility.

### 4.2 Comparison to previous work

In a normal valve, the geometry of the mitral annulus facilitates the filling of the LV in diastole and enables peak tethering forces during ventricular systole to be naturally balanced by distributing stresses generated from increased ventricular pressures toward closure and effective leaflet coaptation. It is reasonable to assume that annular dilatation and diminished annular saddle shape will significantly increase leaflet stress during ventricular systole and diminish the efficacy of coaptation (Salgo et al., 2002). In two in vitro studies using porcine mitral valves, annular dilatation was a prerequisite for significant valvular incompetence, which could not be achieved by ventricular dilatation alone (Espino et al., 2007; He et al., 1997).

Similar to previous descriptions, the mitral area and circularity are at a maximum in mid or late diastole during peak ventricular filling and reach a minimum during mid or late systole in our data (Figure 2). Rausch et al. reported a cyclical change of circularity (described as eccentricity) of 13.5% as compared to 14.2% of maximal fractional change for circularity described herein (Rausch et al., 2011). Also, as previously reported, we observe presystolic MA reduction and tapering of circularity, most likely a consequence of atrial contraction (Glasson et al., 1997; Timek et al., 2003). However, there appears to be more heterogeneity in reports of saddle shape, which could reflect different methods of measurement (Silbiger, 2012; Timek & Miller, 2001). As demonstrated, we observe a more linear rise and fall of NPA, being flattest in late diastole and more saddle‐shaped in late systole (Figure 2). The phasic discoordination of NPA from ACI and MAA emphasizes that annular area reduction is not mediated by a folding of the saddle shape alone but also assumes a sphincteric‐like contraction.

Lacking inherent contractile elements, the motion of the MA is assumed to be secondary to extrinsic forces exerted by atrial and ventricular contraction. One anatomical study described the presence of circumferentially oriented subepicardial muscle fibers at the base of the left atrium and obliquely oriented muscle fibers at the inlet of the LV to be the likely mediators of annular contraction (Silbiger & Bazaz, 2020). This supports the present findings that reduced ventricular contractility results in a diminished MAA contraction (Figure 3).

In a sheep model with implanted markers in the mitral annulus, Carlhäll and colleagues reported reduced MA area with inotropic stimulation (Carlhäll et al., 2007). One animal experimental model using closed chest valvulectomy recognized that the magnitude of MR is not exclusively dependent on atrioventricular gradients, but rather on volume or pressure load (Borgenhagen et al., 1977). Further work in an open chest model by Yoran et al. showed that peripheral vasoconstriction by angiotensin caused increased MR by elevation of transmitral gradients and increased ventricular volumes and regurgitant area. On the contrary, norepinephrine reduced MR, although systolic pressure gradients were substantially increased (Yoran et al., 1979). Although ventricular and annular geometries were not directly measured, the authors concluded that the effect of norepinephrine was due to reduced regurgitant area from increased contractility and reasoned that myocardial contractility is an important mediator of mitral valvular dynamic function. These findings are supported by the current study in which increased afterload by aortic occlusion causes the MA to dilate consistently during the entire cardiac cycle (Figure 2, Table 2). Furthermore, we demonstrate a strong association between myocardial contractility and MA area reduction during the cardiac cycle (Figure 3), which could provide an explanation for MR reduction with norepinephrine infusion.

### 4.3 Clinical inferences

Taking the limitations into consideration, the findings from this study may have implications for our understanding of mitral valve pathophysiology and treatment alike. Although functional MR is commonly associated with annular dilatation and ventricular remodeling, the relative contribution of LV load and contractility to annular dynamics and in extension, MR, remains undetermined.

The vulnerability to functional MR in patients in absence of ventricular enlargement and atrial fibrillation has not been offered a unifying explanation. One possible explanation, as observed in patients with ventricular functional MR treated with cardiac resynchronization therapy, is that an attenuated rise in LV pressure (*d*P/*d*t_max_) following reduced LV contractility may delay mitral valve closure and result in an ineffective coaptation (Breithardt et al., 2003). It is therefore claimed that LV dysfunction prompts MR by reducing valvular closing forces (Asgar et al., 2015). Similar to patients with atrial functional MR, heart failure with preserved EF has been associated with left atrium enlargement and dysfunction, but this finding has not been given full credibility in the causation of the development of MR (Deferm et al., 2019; Kotecha et al., 2016; Tamargo et al., 2020). The present study describes that reduced contractility is associated with impaired MAA and NPA dynamics (Figure 3), presumably impairing coaptation, which may help clarify the susceptibility to MR in patients with ventricular dysfunction and/or annular dilatation in absence of ventricular remodeling.

Furthermore, functional MR has been shown to be dynamic and may substantially worsen in response to exercise and loading conditions (Bertrand et al., 2017; Keren et al., 1989). Interestingly, progressive annular dilatation and reduced MAA contraction during exercise have both been shown to be reliable predictors of dynamic MR deterioration, whereas the degree of MR and annular dilatation at rest, EF, or LV volume has not (Bertrand et al., 2017). Exercise‐induced MR has been shown to be associated with reduced exercise capacity and adverse clinical outcomes, and may be elemental in the development of acute pulmonary edema (Bertrand et al., 2017; Piérard & Lancellotti, 2004). In the present study, significant flattening and dilatation of the MA in response to increased cardiac load becomes evident (Figure 2, Table 2), which may be a contributing factor and explanation for dynamic MR deterioration in this patient group.

Also demonstrated herein, transient inferior caval constriction results in a uniform reduction of the mitral annular area with no change in circularity and saddle shape and a concomitant reduction in end‐diastolic LV volume (Tables 1 and 2). Conceivably, this will reduce both tethering and closing forces of the mitral valve, improving leaflet coaptation. Although not directly applicable to clinical practice, this geometrical change may help explain the benefits of preload‐reducing medications in acute MR, which are conventionally credited to the reduction of transmitral gradients (Keren et al., 1986; Levine & Schwammenthal, 2005; Yoran et al., 1979).

Irrespective of mitral valvular competency, mitral annular velocity measurements are a useful determinant of LV function, which is commonly used in clinical practice. This is based on the assumption that mitral annular longitudinal displacement corresponds to the shortening of longitudinal ventricular muscle fibers (Seo et al., 2010). Based on the presence of obliquely oriented muscle fibers at the inlet of the LV (Silbiger & Bazaz, 2020), comparable associations would be warranted for MA reduction. There is a significant correlation between a load‐independent index of ventricular contractility and average velocity of negative annular deformation in our data (Figure 3), arguably providing additional justification for the rationale of annular velocity measurements as an indicator of LV function.

### 4.4 Computational analyses of anatomical data

Using sonomicrometry array localization with piezoelectric transducers allows for physiologic experiments to be performed with a high degree of temporal and spatial resolution (Gorman 3rd et al., 1996). Nevertheless, sonomicrometry necessitates invasive implantation procedures but may have limited influence on cardiovascular function with careful handling (Korinek et al., 2007).

Conventional analyses utilizing sonomicrometer transducers or radiopaque markers assume perfect anatomical placement. For instance, commissures can be difficult to identify in the arrested animal heart, and calculations of circularity require dimensions at exact orthogonal angles. Small deviations in marker or transducer positions will produce differing results and impair comparability. By applying a hybrid approach with computational analysis, previous methodological limitations can be overcome. In the present study, we have adopted a computational analysis using cubic spline interpolation and principal component analysis that allows precise measurements to be obtained unrestricted of marker or transducer positions. Also, in preference to area calculations by usual Delaunay triangulation in which marker positions are interconnected with straight lines, we advocate the use of a spline interpolation technique representative of a biologic structure. Furthermore, for comparison of data from different cardiac cycles, we encourage that data should be corrected for sampling frequency and heart rate. In the current study, this was performed with linear interpolation, which provides a reliable concatenation of data points considering the high sampling frequency of the sonomicrometric acquisition.

### 4.5 Limitations

First, the experiments were conducted in an open‐chest animal model under general anesthesia, which influences both cardiac and hemodynamic function. Second, the experimental protocol did not include a controlled intervention to increase or reduce LV contractility. Instead, variations of LV contractility were achieved as a result of the cardiodepressant effects from cardiopulmonary bypass and anesthetic agents. Finally, the experiments were performed during acute conditions in a small sample of young animals without any known pathology, for which the translational significance to human physiology should be interpreted with caution.

### 4.6 Conclusion

In conclusion, the present study demonstrates that loading conditions and ventricular contractility influence aspects of mitral annular geometry and deformation, possibly explaining the susceptibility of certain patient populations to acute mitral incompetence.

## AUTHOR CONTRIBUTIONS

R.M.P. planned and executed all the experiments, acquired ethical consent, analyzed data and prepared figures, and wrote the manuscript. H.M.D.A. and V.E.P. performed computational analyses. K.G. and J.P.E.K. aided in the design and execution of experiments and provided guidance and advice in data analysis and writing of the manuscript. P‐R.S. and L.S. provided surgical assistance in animal experiments. B.S. and A.L. performed perfusion on cardiopulmonary bypass in animal experiments. R.H. provided advice in the design and study implementation, as well as in the writing of the manuscript. S.U. was the principal supervisor of the research project and was involved in all parts of the study.

## FUNDING INFORMATION

The work was supported by Western Norway Regional Health Authority and Simon Fougner Hartmann's Foundation.

## CONFLICT OF INTEREST STATEMENT

The authors declare no conflicts of interest relevant to this study.

## APPENDIX A Mitral annular intercommisural width (ICW) and septolateral distance (SL) at baseline, inferior caval constriction (ICC), and aortic occlusion (AO)

### A.1

**Table:**

Variable	Baseline	ICC	AO	RM‐ANOVA
ICW‐ED (mm)	33.5 ± 1.0	32.4 ± 1.2*	34.1 ± 1.0*	*p* < 0.001
ICW‐SP_max_ (mm)	32.9 (30.8; 35.7)	32.1 (30.1; 35.6)*	33.6 (31.6; 36.1)	*p* = 0.001
ICW‐ES (mm)	32.1 ± 1.3	31.6 ± 1.3*	32.8 ± 1.3*	*p* < 0.001
SL‐ED (mm)	23.6 ± 0.9	22.8 ± 0.9	23.7 ± 1.1	*p* = 0.14
SL‐SP_max_ (mm)	23.3 ± 1.0	22.6 ± 1.0	23.8 ± 1.1	*p* = 0.003
SL‐ES (mm)	21.1 ± 1.0	20.4 ± 0.9	21.8 ± 1.1*	*p* = 0.002

*Note*: Values are mean ± SE or median (1st quartile; 3rd quartile); *n* = 10.

Abbreviations: ‐ED, ‐ES and ‐SP_max_, delineators for end‐diastole, end‐systole, and peak systolic left ventricular pressure; ICW, intercommissural width; SL, septolateral distance. *p*, *p*‐values for one‐way repeated measurement ANOVA or Friedman Repeated Measures Analysis of Variance on Ranks.

* Significantly different from baseline with multiple contrast tests.
